# Iontophoretic and Microneedle Mediated Transdermal Delivery of Glycopyrrolate

**DOI:** 10.3390/pharmaceutics6040663

**Published:** 2014-12-22

**Authors:** Meera Gujjar, Ajay K. Banga

**Affiliations:** Department of Pharmaceutical Sciences, Mercer University, 3001 Mercer University Drive, Atlanta, GA 30341, USA; E-Mail: Meera.Suresh.Gujjar@live.mercer.edu

**Keywords:** iontophoresis, microneedles, transdermal, quaternary ammonium, glycopyrrolate

## Abstract

Purpose: The objective of this study was to investigate the use of iontophoresis, soluble microneedles and their combination for the transdermal delivery of glycopyrrolate. Methods: *In vitro* permeation was tested using full thickness porcine ear skin mounted onto Franz diffusion cells. Iontophoresis (0.5 mA/cm^2^) was done for 4 h using Ag/AgCl electrodes. For microneedles, three line array (27 needles/line) of maltose microneedles were used to microporate the skin prior to mounting. Pore uniformity was determined by taking fluorescent images of distribution of calcein into pores and processing the images using an image analysis tool, which measured the fluorescent intensity in and around each pore to provide a pore permeability index (PPI). The donor chamber contained 500 µL of a 1 mg/mL solution of glycopyrrolate, and the receptor chamber contained 5 mL of 50 mM NaCl in deionized water. Samples were collected at predetermined time points over a period of 24 h and analyzed by HPLC. Skin irritation testing was performed with a 3D cell culture kit of human skin. MTT assay determined cell viability; viability less than 50% was considered irritant. Results: A control experiment which investigated passive permeation of glycopyrrolate delivered an average cumulative amount of 24.92 ± 1.77 µg/cm^2^ at 24 h, while microneedle pretreatment increased permeability to 46.54 ± 6.9 µg/cm^2^. Both iontophoresis (158.53 ± 17.50 µg/cm^2^) and a combination of iontophoresis and microneedles (182.43 ± 20.06 µg/ cm^2^) significantly increased delivery compared to passive and microneedles alone. Glycopyrrolate solution was found to be nonirritant with cell viability of 70.4% ± 5.03%. Conclusion: Iontophoresis and a combination of iontophoresis with microneedle pretreatment can be effectively used to enhance the transdermal delivery of glycopyrrolate. Glycopyrrolate was found to be non-irritant to skin.

## 1. Introduction

Anticholinergics are a class of drugs used to treat diseases, such as asthma, chronic obstructive pulmonary disorder (COPD), and peptic ulcers. They are associated with side effects including constipation, dry mouth, and gastric irritation. Glycopyrrolate is an anti-muscarinic agent used in the treatment of excessive secretions. Due to its short half-life and poor bioavailability, glycopyrrolate is administered several times a day. For pediatric patients, several injections a day can be painful and inconvenient. Glycopyrrolate has a quaternary ammonium structure, retaining a positive charge regardless of change in pH, has a molecular weight of 380 Da, and a log *P* of −1.18. The physicochemical properties of this molecule, short half-life, and poor bioavailability make it an ideal candidate for transdermal delivery using physical enhancement techniques.

Transdermal delivery offers the advantages of bypassing first pass metabolism, increased bioavailability, and patient compliance. Studies have been conducted on topically applied glycopyrrolate for gustatory sweating [1], frey’s syndrome [2], and hyperhidrosis [3,4,5]. A small clinical study comparing the transdermal and oral route of delivery for oxybutynin found the transdermal route to have similar efficacy and better side effect profile compared to oral route [6]. The stratum corneum, the outermost layer of the skin, is a rate limiting barrier to permeation of chemicals. For this reason, several active enhancement technologies have surfaced as methods to enhance the scope of drugs which can be delivered transdermally.

Iontophoresis is one such technique that utilizes the application of a physiologically acceptable current and works on the principle of “like repels like”, driving charged molecules through the skin [7]. Microneedles are micron sized needles that breach the stratum corneum, making drugs accessible to the dermis and systemic circulation. Several types of microneedles have been fabricated, such as maltose, metal, polymer, and glass [8]. The microchannels created in the skin are hydrophilic in nature due to the influx of interstitial fluid, and therefore can enhance the delivery of hydrophilic drugs. Due to the hydrophilicity and charged nature of glycopyrrolate, the objective of this study was to assess its transdermal delivery using iontophoresis and microneedles.

## 2. Materials and Methods

### 2.1. Chemicals

Glycopyrrolate was purchased from Sigma Aldrich (St. Louis, MO, USA). HPLC solvents were obtained from Fisher Scientific (Pittsburgh, PA, USA). The irritation kit and MTT assay supplies were obtained from MatTek Corporation (Ashland, MA, USA).

### 2.2. Skin Preparation

Full thickness porcine skin was obtained from a local slaughterhouse (Toccoa, GA, USA). Excess fat was removed and skin was stored at −80 °C. Prior to permeation studies, the skin was allowed to thaw, and cut into appropriately sized pieces for permeation.

### 2.3. In Vitro Permeation Studies

Vertical static Franz-type diffusion cells (PermeGear, Hellertown, PA, USA) were used for the permeation studies. The recirculating water bath system was maintained at 37 °C to bring the skin surface temperature to 32 °C. The receptor compartment was filled with DI water containing 0.1 M NaCl for conductivity and skin was mounted with the stratum corneum side facing up. The skin pieces were equilibrated for 15 min. In the donor compartment, 500 µL of a 1 mg/mL solution of glycopyrrolate in water was added. For iontophoresis, a silver/silver chloride electrode couple was used. Glycopyrrolate is positively charged, thus the anode was placed in the donor compartment. A current of 0.5 mA/cm^2^ was applied for the first 4 h. Maltose microneedles were inserted into the skin for approximately 1 min prior to mounting the skin to allow for them to dissolve. Receptor samples were collected at predetermined time points and analyzed for drug content by HPLC.

### 2.4. Calculation of Lag Time

Lag time was determined by finding the linear portion of the cumulative amount *versus* time plot and extrapolating back to the *x*-axis. A linear regression was obtained and the *y* value was set to zero. Lag time was then calculated by solving for *x*.

### 2.5. HPLC

HPLC analysis was carried out on Alliance HPLC Waters 2695 Separations Module attached to a Waters UV detector. The mobile phase consisted of acetonitrile: 0.1 M ammonium acetate (pH 4.8 with formic acid) (34:66). A Gemini NX C18, 5u 110A, 150 × 4.6 mm column with flow rate of 0.6 mL/min was used. Chromatographic conditions were maintained at room temperature and detection wavelength was set to 230 nm.

### 2.6. Irritation Test

A 3D cell culture model of human keratinocytes was purchased from MatTek Corporation. Irritation testing was performed according to manufacturer’s protocol. Briefly, upon arrival of the kit, fresh media was replaced and tissue inserts were incubated overnight at 37 °C with 5% CO_2_. The next day, tissues were dosed with 30 µL of saline (negative control), 30 µL of 5% sodium dodecyl sulfate (positive control), and 30 µL of glycopyrrolate solution (*n* = 3 for each group). After incubating for 1 h, the surface of the tissues was washed thoroughly with saline solution to remove any residual solution. The tissue inserts were incubated again for approximately 24 h. MTT reagent was added and allowed to incubate for 3 h followed by isopropanol extraction for 2 h. Absorbance was measured at 340 nm. Cell viability was calculated using a spreadsheet provided by MatTek; viability less than 50% was determined to be irritant.

### 2.7. Statistical Analysis

Statistical analysis for multiple groups was carried out using single factor one way ANOVA. Tukey’s test was performed to determine significant difference between the groups. A 0.05 level of probability (*p* < 0.05) was taken as the level of significance.

## 3. Results

### 3.1. In Vitro Permeation with Active and Passive Delivery

**Figure 1:**
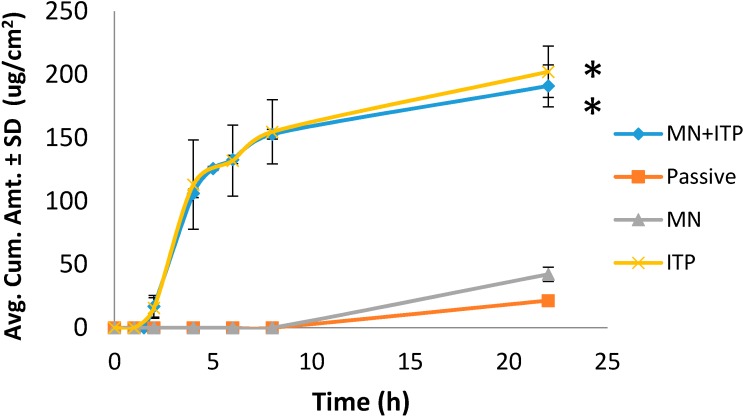
Comparison of glycopyrrolate permeation with passive and active delivery. MN = microneedles, ITP = iontophoresis, MN + ITP = combination of microneedles and iontophoresis. All values represent mean ± SD. * indicates statistically significant compared to passive and MN (*p* < 0.05).

Four methods of delivery were compared: passive, microneedles, iontophoresis, combination of iontophoresis and microneedles. As seen in Figure 1, passive transport resulted in delivering 21.49 ± 1.82 µg/cm^2^ of glycopyrrolate. Poration with microneedles increased delivery to 42.23 ± 9.90 µg/cm^2^. Iontophoresis and combination of iontophoresis with microneedles both significantly increased delivery approximately 10 fold to 202.25 ± 35.30 µg/cm^2^ and 191.04 ± 28.62 µg/cm^2^, respectively. No synergistic effect was observed with combination of iontophoresis and microneedles.

### 3.2. Visualization of Microchannels

**Figure 2:**
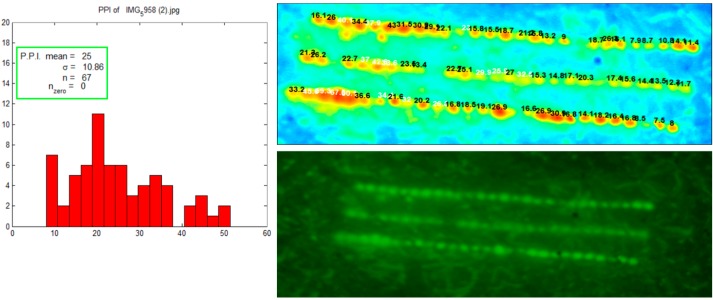
Visualization and uniformity of pores created with microneedles. Calcein fluorescent dye was applied on microporated skin for visualization. Fluropore software generated histogram shows uniformity of pores.

After insertion of maltose microneedles, a calcein fluorescent dye was applied to the skin. Calcein is a hydrophilic dye that diffuses into the aqueous microchannels. Figure 2 images images were immediately taken using a fluorescent camera (Nikon camera integrated with a macrolens and 525 nm long pass filter, Canon Inc, Japan). The images were further analyzed by Fluoropore software which measures fluorescent intensity around each pore and calculates a value called as pore permeability index (PPI). The histogram shows a relatively uniform distribution of pores.

### 3.3. Lag Time

**Figure 3:**
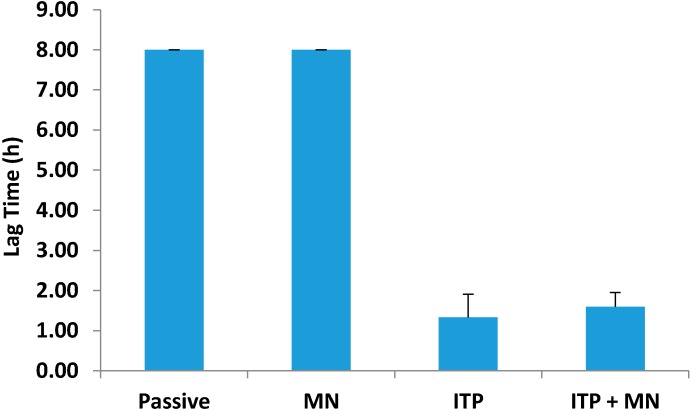
Lag time associated with passive and active delivery. All values represent mean ± SD.

The lag time was measured by extrapolating from the linear portion of the permeation profile back to the *x*-axis. Passive permeation and microneedles had the same lag time of 8.00 ± 0.00 h (Figure 3). Iontophoresis reduced the lag time down to 1.33 ± 0.58 h, suggesting much faster onset of action compared to passive delivery. Combination of iontophoresis and microneedles reduced the lag time to 1.60 ± 0.35 h, but no further reduction in lag time was observed compared to iontophoresis alone.

### 3.4. Assessment of Skin Irritation

**Figure 4:**
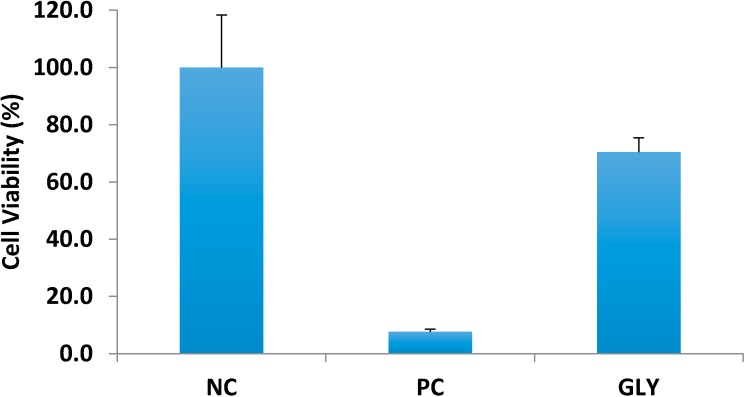
Assessment of glycopyrrolate dermal irritation. NC = negative control (saline), PC = positive control (5% sodium dodecyl sulfate), GLY = glycopyrrolate solution. All values represent mean ± SD.

Irritation testing was performed using a 3D cell culture of human keratinocytes. The results in Figure 4 show that glycopyrrolate solution was found to be nonirritant with cell viability of 70.4% ± 5.03%. The positive control, sodium dodecyl sulfate, resulted in only 7.7% ± 0.89% cell viability.

## 4. Discussion

Glycopyrrolate is an anti-cholinergic agent with poor bioavailability that must be administered several times a day [9]. Its unique quaternary ammonium structure gives the compound a permanent state of ionization, making it positively charged and hydrophilic. Iontophoresis is a method used to enhance skin delivery of ionized molecules and microneedles enhance the delivery of hydrophilic molecules. Few studies have been done on the topical application of glycopyrrolate. Patients who applied topical glycopyrrolate cream [1], lotion [2], or solution [3] had reduction in sweat. Furthermore, patients subjected to bilateral glycopyrrolate iontophoresis had significantly reduced sweat compared to unilateral tap water iontophoresis in the treatment of palmer hyperhidrosis [10]. The objective of this study was to determine the transdermal delivery of glycopyrrolate using these active techniques.

Figure 1 shows the results comparing active and passive delivery methods. Iontophoresis significantly enhanced the delivery of glycopyrrolate compared to passive and microneedle mediated delivery. Skin has a net negative charge and is therefore selective to permeation of cations [11]. Application of an electrode, in this case anode, with the same charge as the drug produces a repulsive force which drives the molecule through skin. In this study, iontophoresis was applied for the first 4 h. After termination of iontophoresis, no further increase in permeation of glycopyrrolate was observed and flux dropped off. Combination of microneedles and iontophoresis did not result in any synergistic effect. Instead, the combination of techniques resulted in a similar permeation profile to that of iontophoresis alone.

Due to the quaternary ammonium structure, glycopyrrolate is ionized regardless of the pH of skin or the pH of the solution. Unionized molecules are more likely to penetrate across cell barriers, although some studies do show penetration of ionized species via shunt pathways and paracellular space. One reason for the lack of synergism may be due to the small surface area of skin porated by microneedles. Since all molecules are ionized, diffusion/permeation may be limited to the microchannels alone, when microneedles alone are used. In contrast, the application of electrical current via iontophoresis covers the entire surface area of skin. This would allow all glycopyrrolate molecules in solution to be exposed to the repelling force of the anode and allow for more permeation. A study with dissolving polymeric microneedles and iontophoresis also found no synergistic effect for delivery of small molecules. However, a synergistic effect was observed for larger molecules. The authors attributed this to the ionized macromolecule delivery being limited to the microchannels alone (~4.5% of skin surface area), compared to the unionized small molecules which could diffuse from microchannels as well as remaining skin surface (~95.5% area). Therefore, combination of iontophoresis and microneedles would be beneficial for molecules which have poor passive permeation [12]. Similarly, another study with ropinirole hydrochloride found greater permeation with iontophoresis alone, and no further enhancement in permeation with combination of active techniques [13]. However, there are also several reports of synergistic effect, such as delivery of methotrexate [14], prochlorperazine edisylate [15], and fluorescein isothiocynate dextrans [16]. The determining factor for synergistic effect of iontophoresis and microneedles appears to depend on the properties of the drug.

One important factor for transdermal delivery systems is the lag time for when a drug can permeate through skin and be absorbed into systemic circulation. A shorter lag time results in a faster therapeutic effect due to availability of the drug at the site of action. Once in the blood, glycopyrrolate binds competitively to muscarinic acetylcholine receptors and reduces secretions. In our study, passive permeation and microneedle mediated permeation both had lag times of 8 h. However, application of iontophoresis significantly reduced the lag time to 1.3 h. Combination of both active techniques did not reduce the lag time any further, suggesting that iontophoresis alone was driving glycopyrrolate into the receptor compartment. For patients needing glycopyrrolate, topical application with iontophoresis is a feasible option for rapid onset of action. Due to the short half-life, iontophoresis can also allow for programmable delivery to maintain therapeutic levels over a period of time [17].

After determining the feasibility of transdermal delivery of glycopyrrolate, we assessed the potential skin irritation using an *in vitro* cell culture model. Quaternary ammonium compounds are present in several antiseptics and detergents and some are associated with ocular and dermal irritation [18]. Cationic quaternary amines have antimicrobial activity by interacting with anionic groups on bacterial cell membranes. Benzalkonium chloride is a widely used disinfectant in hospital settings known to cause contact dermatitis [19]. The FDA requires that all topical and transdermal products are non-irritant. The 3-dimensional cell culture model used in this study consisted of differentiated human keratinocytes that represent skin metabolically and morphologically. A saline solution served as the negative control and 5% sodium dodecyl sulfate served as the positive control. Our findings (Figure 4 shows that glycopyrrolate solution was found to be nonirritant with cell viability of 70.4% ± 5.03%.

In conclusion, the physicochemical properties of glycopyrrolate (hydrophilic and charged), short half-life, low oral bioavailability, and gastric side effects make it an ideal candidate for transdermal delivery using physical enhancement techniques. Iontophoresis and microneedles were used as methods to enhance the delivery across skin, with iontophoresis serving as the best technique compared to microneedles alone or in combination. Finally, glycopyrrolate solution was found to be non-irritant to skin, suggesting transdermal delivery to be a feasible route of administration.
